# Tuberculosis Peritonitis in an Undiagnosed HIV-Positive Patient: A Case Report

**DOI:** 10.7759/cureus.107033

**Published:** 2026-04-14

**Authors:** Riya Gosrani, Manoj Nair

**Affiliations:** 1 General Surgery, University College London Hospital, London, GBR; 2 Surgery, North Middlesex University Hospital/University College London, London, GBR

**Keywords:** acute peritonitis, bowel perforation and peritonitis, damage control laparotomy, disseminated tuberculosis, hiv-positive

## Abstract

This case highlights the multidisciplinary coordination required to manage perforation peritonitis in a young patient with disseminated tuberculosis, undiagnosed human immunodeficiency virus (HIV), and hemophagocytic lymphohistiocytosis (HLH). A 29-year-old woman presented to the hospital at midnight in septic shock, requiring inotropic support. Collateral history revealed a one-year history of significant weight loss. On examination, the patient appeared cachectic with a grossly distended and tender abdomen. CT imaging showed free fluid, locules of free air, and clustered small bowel loops in the mid-abdomen. Following resuscitation in line with the sepsis-6 protocol, the patient underwent emergency laparotomy. Intraoperatively, matted and dusky small bowel loops were observed, along with 2.5 litres of purulent fluid and widespread white nodules on both visceral and parietal peritoneal surfaces. A damage control approach was adopted: the abdomen was extensively lavaged, biopsies were taken, and drains were inserted, while bowel dissection was deliberately avoided to prevent further injury. Cultures later confirmed atypical mycobacteria and polymicrobial flora. Histology demonstrated caseating granulomas, and further testing revealed newly diagnosed HIV with a high viral load and a severely depleted CD4 count. In the postoperative period, the patient developed migrating enterocutaneous fistulas, which were managed through an intestinal failure protocol alongside anti-tuberculosis therapy. A concurrent diagnosis of HLH delayed the initiation of antiretroviral therapy, necessitating a carefully sequenced treatment approach. Despite the high predicted mortality and complexity of presentation, the patient gradually improved, transitioned to oral feeding, regained weight, and resumed full-time work within a year. This case underscores the importance of early damage control surgery, prompt microbiological and histological sampling, and coordinated multidisciplinary care. Lessons from this case may help inform the management of similarly complex presentations in time-critical and resource-constrained settings.

## Introduction

Perforation peritonitis is a life-threatening surgical emergency typically associated with older adults or patients with pre-existing gastrointestinal pathology [1]. Its occurrence in young individuals is rare and should prompt consideration of atypical infections or underlying immunocompromise [2]. This case report describes a critically unwell 29-year-old patient who presented in septic shock and was subsequently diagnosed with disseminated tuberculosis (TB), previously undiagnosed human immunodeficiency virus (HIV), and hemophagocytic lymphohistiocytosis (HLH). The case illustrates the diagnostic complexity, the importance of timely surgical intervention using a damage control approach, and the value of coordinated multidisciplinary team (MDT) management in achieving a favourable outcome despite a predicted high mortality. It also underscores the need to maintain a broad differential diagnosis in young patients with abdominal sepsis and systemic illness.

## Case presentation

A 29-year-old woman was admitted to the hospital in a critically unwell state after being found collapsed at home by her mother. She had experienced several days of vomiting, abdominal distension, constipation, and increasing confusion. Her past medical history included anorexia, and she had experienced significant unintentional weight loss over the preceding months.

She was hemodynamically unstable on arrival, and on examination, she appeared cachectic, and her abdomen was grossly distended and tender. Laboratory investigations (Table 1) demonstrated leukocytosis with neutrophilia and a significantly elevated C-reactive protein.

**Table 1:** Laboratory results on admission CRP: C-reactive protein; eGFR: estimated glomerular filtration rate

Test	Result	Reference range
Heemoglobin	70 g/L	115-165 g/L
White blood cells	12.27 x 10^9^/L	4.0-11.0 x 10^9^/L
Neutrophils	11.42 x 10^9^/L	2.0-7.5 x 10^9^/L
CRP	260 mg/L	<5.0 mg/L
Lactate	6.7 mmol/L	0.6-2.4 mmol/L
eGFR	22 mL/min/1.73 m^2^	>90 mL/min/1.73 m^2^

**Figure 1:**
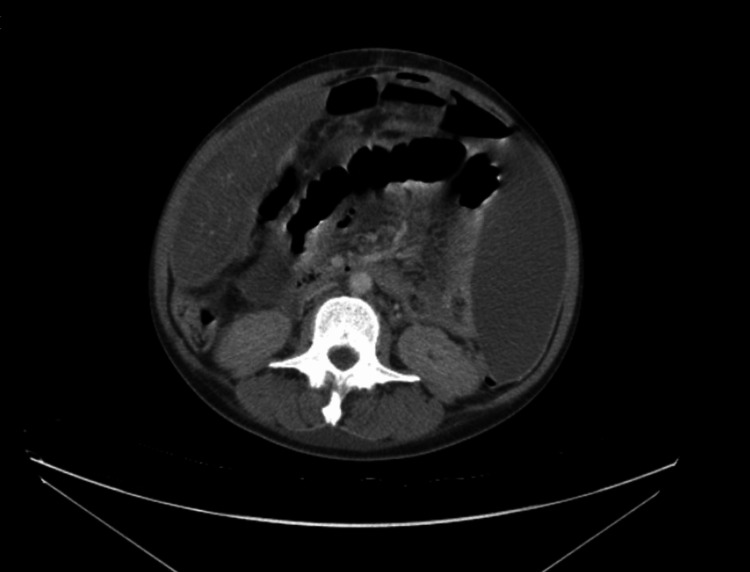
Axial CT - ascites, pneumoperitoneum, and centrally located bowel loops

**Figure 2:**
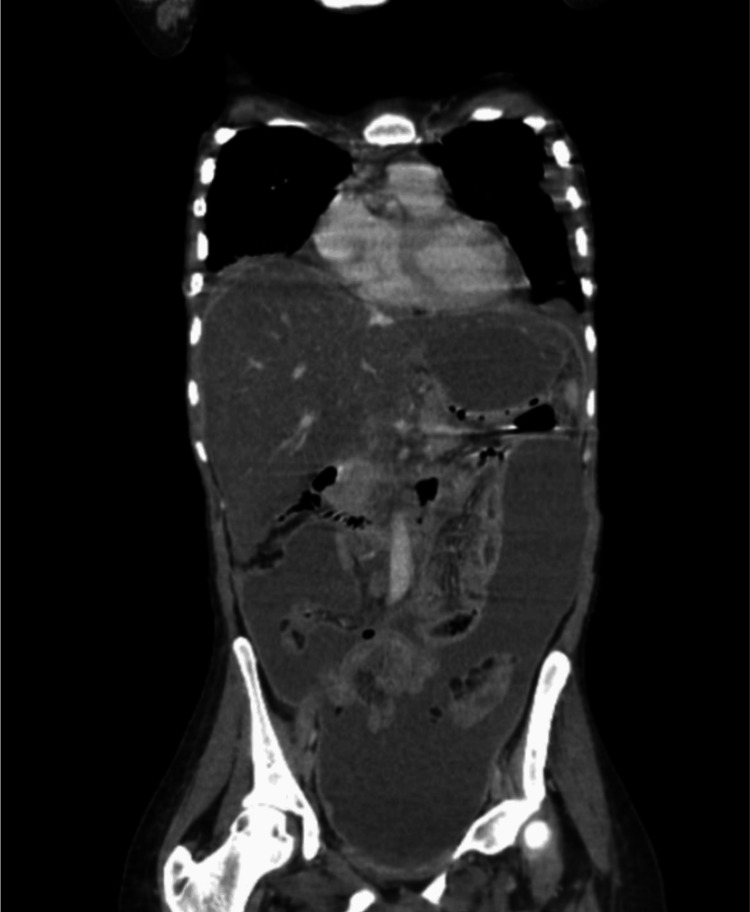
Coronal CT - ascites, pneumoperitoneum, and centrally located bowel loops

Computed tomography scans (Figures 1, 2) demonstrated features consistent with a perforated viscus, including free intraperitoneal gas and fluid, extensive peritoneal thickening, and large-volume ascites. Additional findings included bilateral pleural effusions, splenic nodules, and mediastinal lymphadenopathy. Based on her physiological instability and imaging, the decision was made to proceed with emergency laparotomy following resuscitation under the Sepsis 6 protocol. Her NELA (National Emergency Laparotomy Audit) score predicted a 56.4% mortality risk, while P-POSSUM (Portsmouth Physiological and Operative Severity Score for the enumeration of Mortality and morbidity) predicted 99.9% morbidity [3].

**Figure 3:**
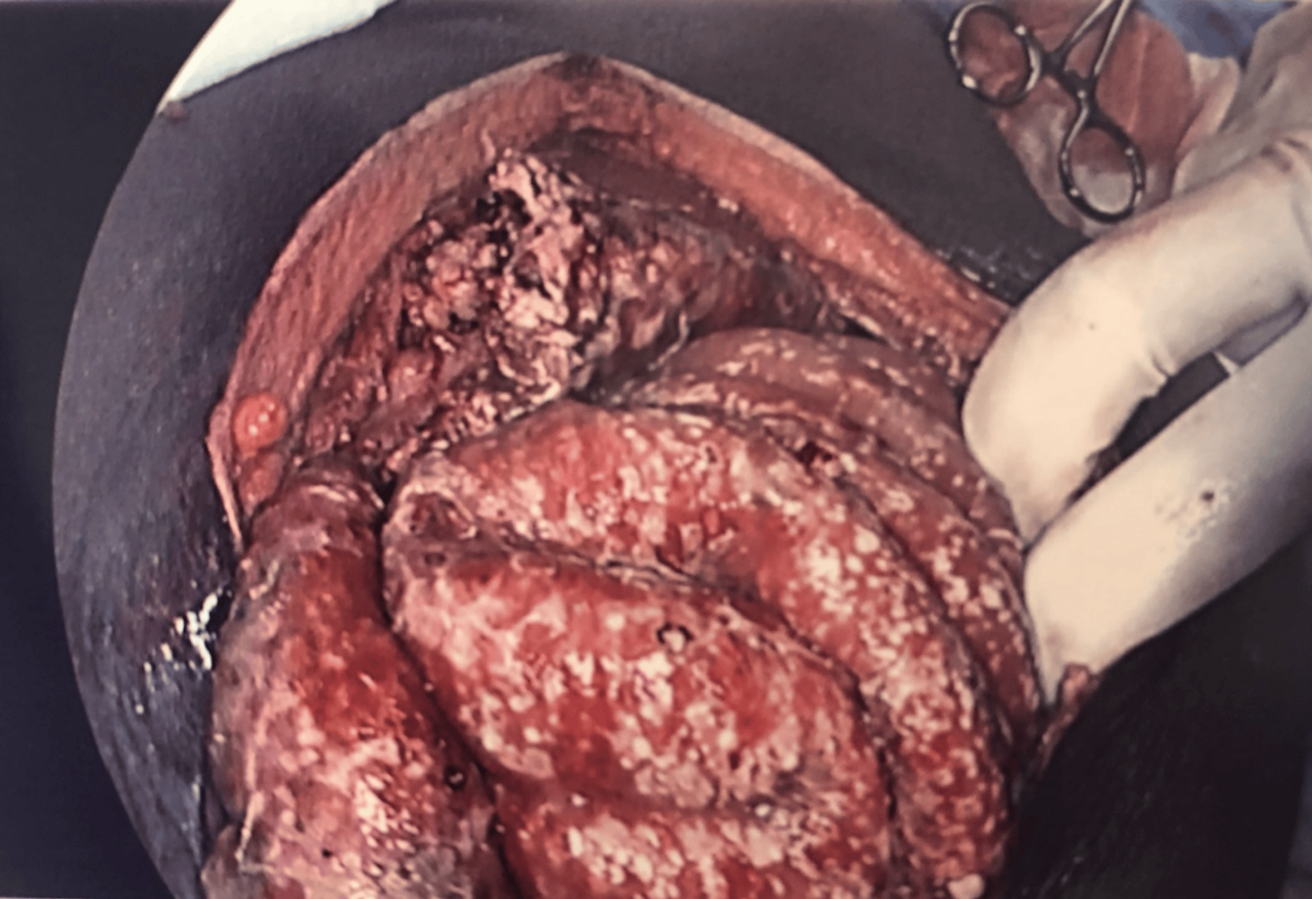
Intraoperative image from laparotomy - miliary TB in the small bowel

**Figure 4:**
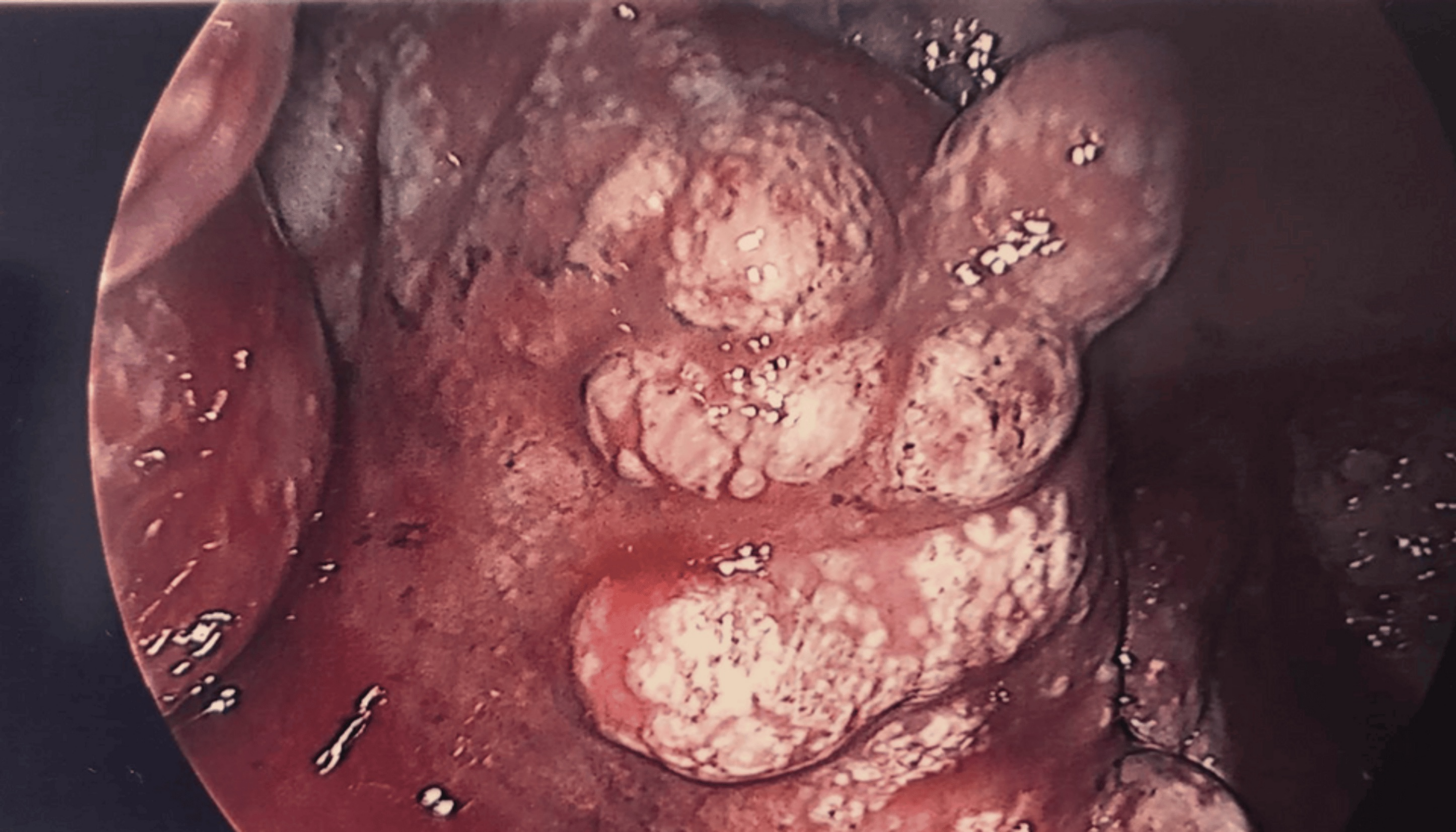
Intraoperative image from laparotomy - miliary TB in the pelvis

Intraoperatively, 2.5 litres of frank pus were drained from the peritoneal cavity. The small bowel was found to be matted, with multiple white nodules visible throughout both the visceral and parietal peritoneum (Figures 3, 4). A damage control approach was adopted to avoid further bowel injury: an extensive peritoneal washout was performed, biopsies were taken, and abdominal drains were inserted. No bowel resection or anastomosis was attempted at this stage.

**Figure 5:**
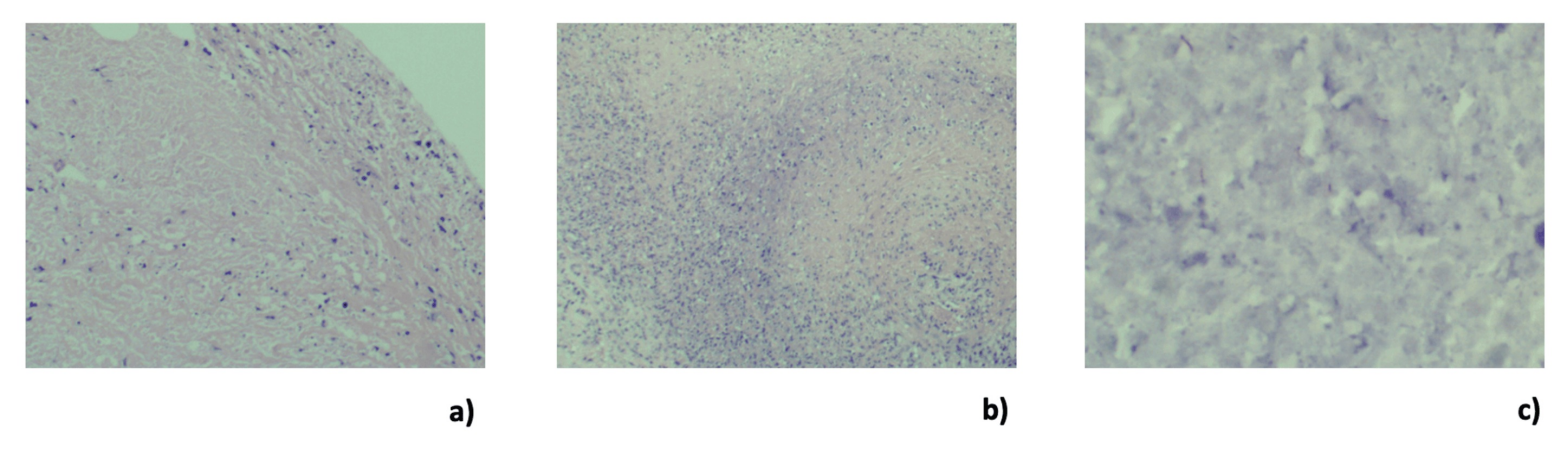
Histopathological images (a) Caseous necrosis; (b) necrotising inflammation; and (c) acid-fast bacilli Ziehl-Neelsen staining

Microbiological analysis of peritoneal and pleural fluid confirmed Mycobacterium tuberculosis via polymerase chain reaction (PCR). Histopathological analysis of intraoperative biopsies demonstrated caseating granulomatous inflammation (Figure 5). The patient was transferred to the intensive care unit, where she required multi-organ support, including mechanical ventilation, inotropes, and continuous haemofiltration. She was commenced on quadruple anti-TB therapy. Over the following days, she developed disseminated intravascular coagulation, and further investigation revealed markedly elevated ferritin and triglyceride levels, raising suspicion for HLH. Treatment with corticosteroids and anakinra was initiated empirically.

Subsequent testing confirmed a new diagnosis of HIV, with a CD4 count of 40 cells/μL and high viral load. Given the risk of immune reconstitution inflammatory syndrome (IRIS), antiretroviral therapy (ART) initiation was deferred and commenced approximately two months after starting anti-TB therapy.

During her recovery, the patient developed multiple migrating enterocutaneous fistulas originating from the laparotomy wound. These were managed conservatively in accordance with an intestinal failure protocol. Following the establishment of both ART and anti-TB treatment, the fistulas gradually closed. Her recovery was supported by regular MDT meetings involving infectious diseases, surgery, intensive care, nutrition, and HIV services.

Over a 12-month period, she transitioned from parenteral to oral feeding, regained weight, and achieved immune reconstitution, with her CD4 count rising above 250 cells/μL within five months. Her HIV viral load became undetectable. Four years after her initial presentation, she remains clinically well, is fully independent, and has returned to full-time employment.

## Discussion

This case illustrates the complexity of managing life-threatening abdominal sepsis in a young, immunocompromised patient with previously undiagnosed HIV and disseminated TB. The use of a damage control surgical approach, in conjunction with rapid resuscitation and coordinated MDT care, was crucial in ensuring survival in the face of overwhelming sepsis, multi-organ dysfunction, and a predicted high mortality risk.

Although peritonitis secondary to intestinal TB is relatively rare [4], it is endemic among immunocompromised patients. In this case, the presentation was acute and fulminant, resembling hollow viscus perforation, but without a discrete perforation site identified intraoperatively. The presence of matted bowel, purulent peritoneal fluid, and widespread nodularity is characteristic of disseminated TB [5] and should prompt immediate biopsy and mycobacterial testing. The diagnosis was further complicated by the presence of HLH - a hyperinflammatory syndrome known to be triggered by infections including TB and HIV [6]. Early recognition of HLH based on laboratory markers such as elevated ferritin and triglycerides allowed timely immunomodulatory therapy with corticosteroids and anakinra [7], which likely contributed to clinical stabilization.

The patient’s profound immunosuppression, reflected by a CD4 count of 40 cells/μL, also placed her at high risk of opportunistic infections and complications. In such cases, the timing of ART initiation is critical. Delayed initiation allowed for control of TB-related inflammation and reduced the risk of IRIS, a paradoxical inflammatory reaction that can worsen clinical outcomes if ART is started prematurely [8].

Damage control laparotomy was pivotal in this case. Avoiding definitive bowel surgery at the index operation reduced the risk of iatrogenic injury in an already friable and inflamed peritoneum. The subsequent development of enterocutaneous fistulas was managed conservatively, with supportive measures guided by intestinal failure protocols and nutritional support. This highlights the importance of ongoing surgical restraint, even in the face of complications, when treating immunocompromised patients with impaired healing capacity.

This case reinforces the need to maintain a high index of suspicion for atypical infections in young patients presenting with abdominal sepsis, especially in the presence of cachexia, weight loss, or systemic features. It also underscores the value of early broad-spectrum diagnostic sampling, including histology and mycobacterial PCR. The MDT plays a central role in managing complex presentations, enabling swift decision-making and improved surgical planning [9]. Collaborative MDT input from infectious diseases, surgery, critical care, HIV specialists, and nutrition services was essential to navigate the complex and evolving clinical picture.

Ultimately, despite multiple adverse prognostic indicators, the patient achieved full recovery and returned to work, a testament to the effectiveness of coordinated care and staged decision-making. This case may offer a useful model for managing similar presentations in resource-limited settings, where delayed diagnosis of HIV and TB remains common, and where surgical decision-making must be both judicious and flexible.

## Conclusions

This case illustrates the role of early damage control surgery, comprehensive microbiological and histopathological evaluation, and multidisciplinary collaboration in the management of abdominal sepsis in an immunocompromised patient. In the setting of previously undiagnosed HIV, disseminated tuberculosis, and secondary HLH, a staged and adaptable approach to treatment was adopted. The patient’s recovery - despite a high predicted risk of mortality - followed sequential therapy for tuberculosis, HIV, and HLH, alongside conservative management of complications such as enterocutaneous fistulas.

Although limited by the nature of a single case, this report highlights the importance of considering underlying immunodeficiency in patients presenting with severe systemic illness and reflects the potential value of a pragmatic, individualised approach in complex clinical scenarios.
